# Lactate and stepwise lactate kinetics can be used to guide resuscitation

**DOI:** 10.1186/s13054-017-1859-y

**Published:** 2017-10-27

**Authors:** Xiang Zhou, Dawei Liu, Longxiang Su

**Affiliations:** Department of Critical Care Medicine, Peking Union Medical College Hospital, Peking Union Medical College & Chinese Academy of Medical Sciences, Beijing, 100730 China

We thank Dr. Thomas-Rüddel for their interest in our study of stepwise lactate kinetics-oriented hemodynamic therapy. Of course, we did not find any difference between the use of inotropes and blood transfusions in our study [1]. However, the average baseline ScvO_2_ in our study was 72.1%, a value quite similar to that of the ProCESS, ARISE, and ProMISe studies [2–4]. Therefore, a large proportion of patients in the ScvO_2_ group (approximately 69% of the patients in our study) could not receive future resuscitation according to the protocol, even though the average enrollment lactate levels were around 5.5 mmol/L. However, the patients with high lactate levels could still enter the lactate kinetics group. An optimal resuscitation target should be able to generate sufficient driving force for the specific clinical treatment so that it can better guide the clinical therapy. Lactate, which reflects tissue hypoperfusion, has been recommended as a valuable resuscitation parameter by new Surviving Sepsis Campaign guidelines [5, 6]. Lactate kinetics, defined by Vincent [7, 8], represent a balance between lactate production and elimination and can represent a resuscitation parameter [9, 10]. In our study, it was obvious that, compared with ScvO_2_, lactate kinetics could not only support a proper initial resuscitation, but could also guide the whole therapeutic process.

Although the lactate kinetics group were more actively resuscitated compared with the ScvO_2_ group, these patients received a more restrictive fluid regimen. Provided tissue perfusion was sufficient, we followed the principle of the lower the central venous pressure (CVP), the better the outcome. Many studies have demonstrated lower CVP associated with better outcome in sepsis [11, 12]. Thus, after the initial resuscitation, as the lactate kinetics targets were achieved, we tried to keep the CVP as low as possible. Even then, the lactate kinetic group still needed more fluid and had higher CVP. But compared with the trial by Jansen et al. [13], fewer crystalloid fluids were infused at each time-point, and more red blood cells were transfused in the whole process in our study. We are not sure whether this is related to the patient's prognosis. The main purpose of this stepwise lactate kinetics target-oriented therapy was to achieve effective resuscitation while avoiding overtreatment.

We strictly followed the protocol, and a patient who received the treatment of the other group was still assigned to the first group, by intention-to-treat analysis. So we did not have lactate kinetics data for the ScvO2 group.

As a single-center randomized controlled trial, fragility index had little influence on our study. The sample size was appropriate for both inferiority and non-inferiority trials. Additionally, we did try to strictly follow the study protocol to minimize bias. Blind testing was also used throughout the whole process of this study.

In our opinion, lactate is an important parameter for monitoring tissue perfusion at present. Lactate kinetics are particularly important to evaluate the response of ICU patients. Therefore, lactate can be the starting point for resuscitation, and monitored during the process of resuscitation to assess whether tissue perfusion is being adequately restored.
